# Cold Plasma Treatment Enhances Drought Tolerance of Alfalfa (*Medicago sativa* L.) Seeds by Modulating Physiological Responses and Transcriptomic Profiles

**DOI:** 10.3390/antiox15060681

**Published:** 2026-05-28

**Authors:** Weicheng Gong, Chunxu Qin, Zhiqing Song, Xiliang Hao, Aozhe Li, Yaxin Liu, Chengzhi Ma

**Affiliations:** 1 College of Electric Power, Inner Mongolia University of Technology, Hohhot 010080, China; 202311211352@imut.edu.cn (W.G.); qincx@imut.edu.cn (C.Q.); 202311211019@imut.edu.cn (X.H.); 202311211392@imut.edu.cn (A.L.); 202311211332@imut.edu.cn (Y.L.); 202311211372@imut.edu.cn (C.M.); 2 Inner Mongolia Key Laboratory of Intelligent Control for New Energy Power Systems, Hohhot 010080, China; 3 Engineering Research Center of the Ministry of Education for Large Scale Energy Storage Technology, Hohhot 010080, China

**Keywords:** alfalfas (*Medicago Sativa* L.), cold plasma, drought stresses, antioxidant enzymes, transcriptomes

## Abstract

Drought stress is a major limiting factor for alfalfa (*Medicago sativa* L.) production in arid and semi-arid regions. Cold plasma treatment has emerged as a promising physical technology for improving seed germination and stress tolerance, but its underlying mechanisms remain poorly understood. In this study, alfalfa seeds were treated with cold plasma (plasma discharge voltage: 0, 5, 10, 15 kV) for 5 min and exposed to PEG 6000 stress at 0, 5, 10, and 15%. Results showed that cold plasma treatment significantly alleviated the inhibitory effects of drought stress on seed germination, with the Plasma-15 kV treatment exhibiting the highest germination potential and germination rate compared to the control (*p* < 0.05). Plasma treatment enhanced the activities of superoxide dismutase (SOD), peroxidase (POD), and catalase (CAT), while reducing malondialdehyde (MDA) content (*p* < 0.05), indicating mitigated oxidative damage under drought conditions. Transcriptomic analysis revealed that cold plasma regulated the expression of genes involved in the MAPK signaling pathway and other drought-responsive pathways, leading to metabolic reallocation (*Q* < 0.05) and enhanced drought tolerance. In conclusion, 5 min of Plasma-15 kV treatment effectively enhances drought tolerance via physiological and transcriptional regulation, providing an eco-friendly strategy for alfalfa cultivation in dry regions.

## 1. Introduction

Drought stress is one of the most serious abiotic stresses in global agricultural production, which seriously restricts the growth and development [1,2], yield formation [3,4,5,6,7], and ecological stability of crops [8,9]. As global climate change worsens, its negative impact continues to expand [10]. Therefore, exploring efficient and environmentally friendly crop drought resistance improvement technology has become a hot topic in current agricultural research [11,12,13]. Alfalfa (*Medicago sativa* L.), a globally recognized high-quality legume forage, has high protein content and good palatability. It is the core forage resource for the development of animal husbandry, and plays a key role in ecological restoration, such as soil and water conservation and soil improvement [14,15]. However, the seedling stage and growth period of alfalfa are highly sensitive to water deficit. Drought stress can significantly inhibit seed germination, seedling growth, biomass accumulation, and root development, resulting in slow growth and a significant decline in yield and quality [16,17,18]. This problem has become a key factor restricting the large-scale and sustainable development of the alfalfa industry in arid and semi-arid areas. The traditional drought resistance improvement methods of the alfalfa, mainly including conventional selective breeding [19], exogenous hormone spraying [20], as well as water and fertilizer regulation [21], have obvious limitations, such as a long breeding cycle, easy environmental residue caused by exogenous hormone application, high cost of water and fertilizer regulation, and limited effect due to natural conditions. Therefore, it is urgent to develop a green, efficient, and easy-to-operate physical regulation technology to enhance alfalfa’s drought adaptability by activating its inherent stress resistance.

Cold plasma technology, as a new type of agricultural physical pretreatment technology, has shown significant advantages in seed germination regulation [22,23] and plant stress resistance improvement [24]. It exerts mild stimulation on crops through compound fields such as reactive oxygen nitrogen species, ultraviolet light, and electric field to regulate the plant growth process [25,26], and induces stress resistance gene expression [27], which conforms to the basic principle of plants responding to external drought stress [28,29,30]. At present, this technology has been successfully applied in grain crops such as wheat [31,32,33], oat [34,35,36], rice [37,38,39,40], corn [41,42,43], and economic crops such as tomato [44,45,46,47] and rapeseed (*Brassica napus* L.) [48], which provides an important theoretical reference and technical support for its application in the regulation of stress resistance of forage crops.

At present, the research on drought resistance of alfalfa mainly focuses on genetic modification [12,49] and regulation of exogenous substances [13,50]. With the publication of the alfalfa genome and the development of sequencing technology, Previous studies have analyzed the effect of drought stress on alfalfa at the gene level through genome-wide association analysis [51,52]. Studies have confirmed that discharge plasma can increase the germination rate of alfalfa seeds [53]. However, its physiological, biochemical, and molecular regulation mechanisms under drought stress are still unclear, and physiological, biochemical, and molecular levels have not been explored in combination with drought stress scenarios. At the same time, there is no clear conclusion on how cold plasma regulates the antioxidant enzyme system and osmotic adjustment substance synthesis of alfalfa under drought stress, which also limits the practical application of this technology in drought-resistant cultivation of alfalfa.

From the perspective of plant physiological and biochemical responses to drought stress, malondialdehyde (MDA) content and antioxidant enzyme activities are crucial physiological indicators for evaluating plant drought tolerance [54]. MDA is a key product of membrane lipid peroxidation, which can reflect the degree of cell membrane damage induced by drought stress. Antioxidant enzymes, including superoxide dismutase, peroxidase, and catalase, can effectively eliminate excessive reactive oxygen and nitrogen species in plants, maintain the balance of intracellular reactive oxygen metabolism, and reduce oxidative damage under water deficit [55,56]. Therefore, analyzing the changes of these physiological and biochemical indices is essential to reveal the physiological adaptation mechanism of alfalfa to drought stress, and it is also an important theoretical basis for exploring the regulatory effect of cold plasma on crop stress resistance.

Based on this, in this study, alfalfa was used as the experimental material, combined with PEG-6000 simulated gradient drought stress, to explore the effects of different voltage cold plasma treatment on seed germination, seedling growth, physiological and biochemical, and drought resistance gene expression, and to screen the suitable cold plasma treatment voltage to improve its drought resistance. At the level of gene expression, the mechanism by which cold plasma treatment regulates the drought resistance of alfalfa was preliminarily analyzed, providing a green and efficient physical pretreatment technology for drought-resistant cultivation of alfalfa in arid and semi-arid areas.

## 2. Materials and Methods

### 2.1. Experimental Materials and Instruments

The tested alfalfa variety was Zhongmu No.1. The seeds were provided by Jihong Xie, an associate researcher at the Institute of Grassland Research of the Chinese Academy of Agricultural Sciences. The seed purity was ≥98%, and the germination rate was ≥85%. Polyethylene glycol-6000 (PEG-6000, purity ≥ 99%) was provided by Shanghai Guoyao Group, Shanghai, China. Physiological and biochemical indicators determination kits (superoxide dismutase SOD (catalog number: XZK-C-A500), peroxidase POD (catalog number: XZK-C-A502), malondialdehyde MDA (catalog number: XZK-C-A401), catalase CAT (catalog number: XZK-C-A501)) were purchased from Shanghai Xuanze Kang Biological Co., Ltd., Shanghai, China.

**Figure 1:**
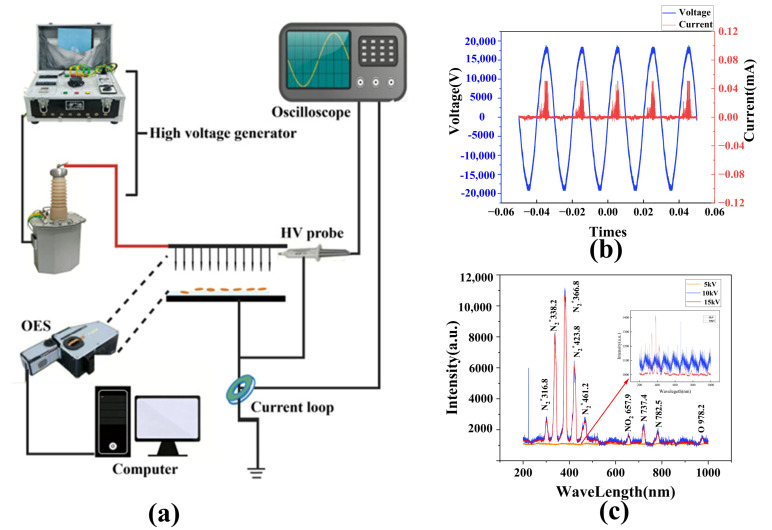
Plasma device and plasma discharge diagnosis. (**a**) Plasma generation and diagnostic device; (**b**) waveform diagram of plasma discharge current and voltage; (**c**) plasma emission spectra.

A needle-array-plate device was used in this study (Figure 1a). The power supply of the experimental device was Alternating Current (AC). The frequency was 50 Hz, and the voltage was continuously adjustable from 0 to 50 kV. The high-voltage electrode comprised a needle array with a length of 2 cm, a needle diameter of 1.56 (±0.02) mm, a curvature radius of 0.75 mm, and horizontal and vertical spacing of 4 cm (14 × 7 needles). The grounding end was a 2 mm-thick planar aluminum plate (85 × 45 cm^2^) covered with a 4 mm-thick Polymethyl Methacrylate (PMMA) dielectric plate (100 × 60 cm^2^). The plasma discharge process was carried out under the conditions of voltage of 5, 10, and 15 kV, and the distance between the tip and the dielectric plate was 3 cm. The applied voltage to the electrodes was monitored by a high-voltage probe (Tektronix P6015A (Tektronix, Beaverton, OR, USA), bandwidth 75 MHz), and the discharge current was measured by a current probe (Pearson Model 6600 (Pearson Electronics, Palo Alto, CA, USA), bandwidth 120 MHz). A digital oscilloscope (Tektronix DPO4104 (Tektronix, Beaverton, OR, USA), bandwidth 1 GHz, sampling frequency 5 GS·s^−1^) was used to record and display all these signals. The waveform of current and voltage in cold plasma discharge is shown in Figure 1b. Plasma emission spectra under different voltages were analyzed using a spectrometer (Kymera Andor 328i, Andor Technology, Belfast, UK) to diagnose the variety and concentration of particles in the plasma produced by the needle-array-plate (spectrometer focal length: 328 mm; image resolution: 0.44–0.31 nm continuously adjustable; detector type: DH334T-16F-E3). The emission spectrum of cold plasma discharge is shown in Figure 1c.

### 2.2. Experimental Design

The two-factor design was used in the experiment, and the plasma discharge voltage was the only experimental factor to determine the optimal voltage level. Four treatment levels of 0 (CK), 5, 10, and 15 kV were set up, and the seed plasma treatment time of all treatment groups was fixed at 5 min. Each voltage treatment group was set up at 0% (deionized water, no drought stress), 5% (mild drought stress), 10% (moderate drought stress), and 15% (severe drought stress) of PEG-6000 simulated drought stress conditions; test group details are shown in Table 1. There were three biological replicates per treatment, and 80 healthy, full-size, uniform seeds were selected for each replicate.

### 2.3. Determination of Seed Germination Index

Seed pretreatment: full and consistent alfalfa seeds were selected, disinfected with 1% Javelle water for 10 min, rinsed with sterile water three times, dried on filter paper, and treated in cold plasma generators at different discharge voltages for 5 min. After treatment, the seeds were placed in a 9 cm culture dish with three layers of filter paper, and 5 mL of PEG-6000 solution at the corresponding concentration was added to each dish, which was then placed in an artificial climate incubator. The culture conditions were a temperature of 24 °C, light intensity of 3000 lx, a light cycle of 14 h/10 h (light/dark), supplementing the corresponding concentration of PEG-6000 solution every 24 h, keeping the filter paper moist and avoiding water accumulation, and avoiding conditions affecting seed germination.

From the second day of culture onward, germinations were counted daily. The germination standard was that the root length is not shorter than the seed length, and the bud length is not shorter than 50% of the seed length. The specific germination indicators were as follows:

(1) $$G_{p}\;=\;\frac{n_{3}}{n}\;\times \;100\%$$

In Equation (1), $G_{p}$ is the germination potential, %; $n_{3}$ is the number of germinated seeds on the third day of the experiment; n is the total number of test seeds.

(2) $$G_{R\;}=\frac{n_{7}}{n}\;\times \;100\%$$

In Equation (2), $G_{R}$ is the germination rate, %; $n_{7}$ is the number of germinated seeds on the 7th day of the experiment; n is the total number of test seeds.

(3) $$G_{I}=\sum \frac{n_{t}}{D_{t}}$$

In Equation (3), $G_{I}$ is the germination index; $n_{t}$ is the number of germinated seeds on the nth day; $D_{t}$ is the try Day tth day of the test.

(4) $$P_{I}=1.0\;\times \;G_{R1}+0.75\;\times \;G_{R3}+0.50\;\times \;G_{R5}+0.25\;\times \;G_{R7}$$

In Equation (4), $P_{I}$ is the germination index; $G_{R1},\;G_{R3},\;G_{R5},\;G_{R7}$ are the germination rate of the seeds on the 1st, 3rd, 5th, and 7th day, respectively, %.

(5) $$V_{I}=P_{I\;}\times \;S_{L}$$

In Equation (5), $V_{I}$ is the vigor index; $P_{I}$ is the germination index; $S_{L}$ is the average length of seedlings on the 7th day of germination, cm.

### 2.4. Determination of Physiological and Biochemical Indexes of Seedlings

The seedlings cultured to the 10th day were selected, and their fresh root tissue was taken. Three biological replicates were set for each treatment. Each replicate was accurately weighed at 0.1 g and quickly placed in a precooled mortar for preparation of root tissue samples. Pre-cooled 0.05 mol·L^−1^ phosphate buffer solution (PBS, pH 7.8) of 1 mL was added to the mortar, and the mortar was fully ground to a homogeneous state under ice bath conditions; the prepared homogenate was transferred to a centrifuge tube and centrifuged at 4 °C, 8000× *g* for 10 min. After centrifugation, the supernatant was carefully drawn and transferred to a new sterile centrifuge tube. The supernatant was the crude enzyme extract, and the MDA content and POD, SOD, and CAT activities were measured according to the kit instructions. Among them, MDA content was determined by the TBA method, POD activity was determined by the guaiacol method, SOD activity was determined by the NBT method, and CAT activity was determined by the ultraviolet absorption method [57]; absorbance was read at 532 nm for MDA (corrected by 600 nm), 470 nm for POD, 560 nm for SOD, and 240 nm for CAT, and samples were not diluted.

### 2.5. Transcriptome Sequencing

Three groups of samples were selected for sequencing: CK1 (no stress without treatment), CK3 (10% PEG stress without treatment), and C2 (10% PEG stress + Plasma 15 kV treatment). Because 5% PEG was mild drought stress, the effect was not obvious; 15% PEG was severe drought stress, and seedlings grew slowly; 15 kV was the optimal treatment voltage screened in the early stage. The Fragments Per Kilobase of transcript per Million mapped reads (FPKM) method was used to calculate gene expression, and differentially expressed genes were screened with |log2(FoldChange)| ≥ 4.5 and *p*-adjust < 0.05 as thresholds. KEGG pathway and GO function enrichment analyses were performed on the differentially expressed genes, and qRT-PCR was used to verify the highly expressed genes related to drought resistance.

### 2.6. Data Processing and Analysis

Statistical analysis was performed using IBM SPSS 27.0 software (IBM, Armonk, NY, USA). One-way analysis of variance was used, and the Duncan post hoc test was used for multiple comparisons. The significance level was set as *p* < 0.05. Origin 2024 software (OriginLab, Northampton, MA, USA) was used to generate charts.

## 3. Results

### 3.1. Effects of Different Voltage Cold Plasma Treatments on Growth Indexes of Alfalfa

**Figure 2:**
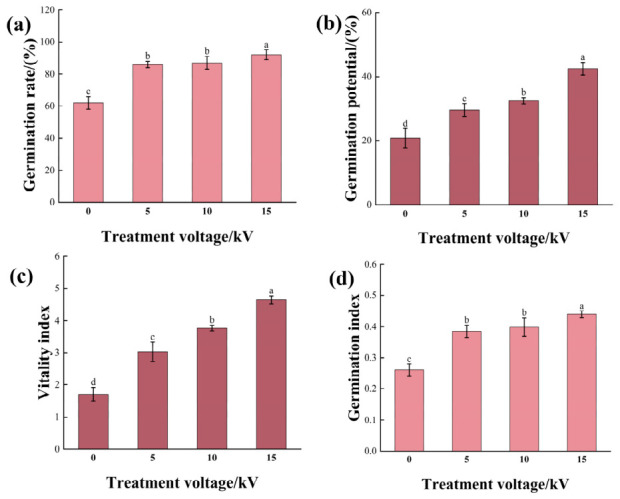
Effects of cold plasma of different discharge voltage treatment on the seed germination index of alfalfa under 10% PEG stress. (**a**) Germination rate; (**b**) Germination potential; (**c**) Vitality index; (**d**) Germination index. Different letters indicate significant differences (*p* < 0.05) between sample means.

The calculation Equations (1)–(5) were used to calculate the germination index of alfalfa seeds under different drought stress and plasma of different discharge voltages. As shown in Figure 2a–d. On the 7th day of incubation, the germination rate, germination potential, vitality index, and germination index of seeds in each cold plasma treatment group were improved to varying degrees compared with the 0 kV control group (*p* < 0.05). Among them, the growth index of the 15 kV cold plasma treatment group was the most significant (*p* < 0.05). The germination rate, germination potential, vigor index, and germination index of seeds were increased by 32.09%, 56.74%, 63.60%, and 40.87%, respectively, in the 15 kV experimental groups compared with the 0 kV control groups.

### 3.2. Effects of Different Voltage Cold Plasma Treatments on Physiological and Biochemical Indexes of Alfalfa

#### 3.2.1. Malondialdehyde (MDA)

**Figure 3:**
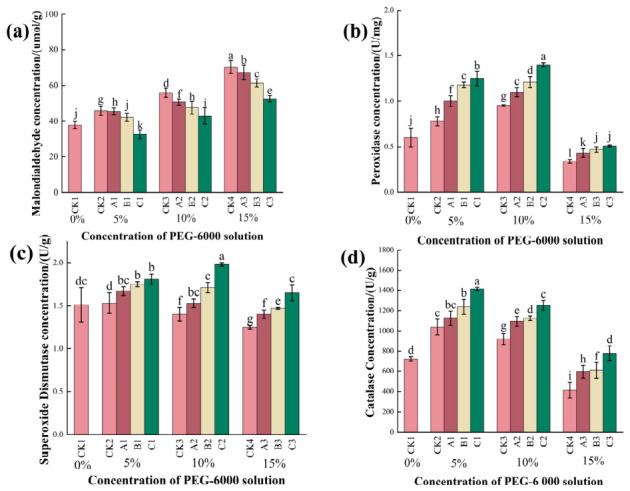
Effects of different discharge-voltage cold plasma treatments on physiological and biochemical indices of alfalfa under different intensities of drought stress. (**a**) Malondialdehyde; (**b**) peroxidase; (**c**) superoxide dismutase; (**d**) catalase. Different letters indicate significant differences (*p* < 0.05) between sample means.

Under the stress of different concentrations of PEG-6000, the changes of MDA content in plants with the increase of plasma treatment discharge voltage are shown in Figure 3a. Compared with the CK2, CK3, and CK4 groups, the MDA content in the cold plasma treatment groups with different discharge voltages was lower than that of the control group, and it decreased as the discharge voltage increased. Under the cold plasma treatment of 15 kV, the MDA activities of C1, C2, and C3 groups were 32.56 μmol/g, 42.88 μmol/g and 52.46 μmol/g, respectively, which were 21.95%, 16.28%, and 29.59% lower than those of CK2, CK3, and CK4 groups, respectively. The above results showed that cold plasma treatment could relatively reduce the degree of lipid peroxidation and membrane damage of alfalfa under drought stress.

#### 3.2.2. Peroxidase (POD)

Under different concentrations of PEG-6000 stress, the changes of POD activity in plants with the increase of plasma discharge voltage are shown in Figure 3b. Compared with the CK2, CK3, and CK4 groups, the POD activity of discharge plasma-treated plasma groups at different discharge voltages was higher than that of the control group, and the POD activity showed an overall upward trend with increasing treatment voltage. Under the treatment of 15 kV cold plasma, the POD activities of C1, C2, and C3 groups were 1.09 U/mg, 1.22 U/mg and 0.6 U/mg, respectively, which were 37.6%, 44%, and 23.91% higher than those of CK2, CK3, and CK4 groups. The above results showed that cold plasma treatment could effectively improve the POD activity of alfalfa seedlings, thereby reducing the oxidative damage caused by drought stress.

#### 3.2.3. Superoxide Dismutase (SOD)

Under different concentrations of PEG-6000 stress, the changes in SOD activity in plants with the increase of plasma discharge voltage are shown in Figure 3c. Compared with the control group, plasma treatment at different discharge voltages could significantly increase the SOD activity of seedlings, and the SOD activity increased with the increase in treatment voltage. At 15 kV, the SOD activities of the C1, C2, and C3 groups were 1.81 U/g, 1.98 U/g, and 1.75 U/g, respectively, which were 15.47%, 29.29%, and 27.43% higher than those of the CK2, CK3, and CK4 groups, respectively. The above results showed that cold plasma treatment could increase SOD activity in plants, strengthen the antioxidant system to remove excess reactive oxygen species, and reduce lipid peroxidation damage to membranes.

#### 3.2.4. Catalase (CAT)

Under different concentrations of PEG-6000 stress, the changes in CAT activity in plants with the increase of plasma discharge voltage are shown in Figure 3d. Compared with CK2, CK3, and CK4, different discharge-voltage plasma treatments could increase the CAT activity of seedlings, and the CAT activity increased with the increase of discharge voltage. Under the condition of 15 kV, the CAT activities of C1, C2, and C3 groups were 1412.95 U/g, 1252.4 U/g, and 777 U/g, respectively, which were 26.65%, 26.70%, and 46.83% higher than those of CK2, CK3, and CK4 groups, respectively. The above results showed that cold plasma treatment could increase CAT activity in plants, enhance the alfalfa’s scavenging ability of hydrogen peroxide, and alleviate the damage caused by reactive oxygen species to cell structure and physiological function.

### 3.3. Gene Differential Expression

**Figure 4:**
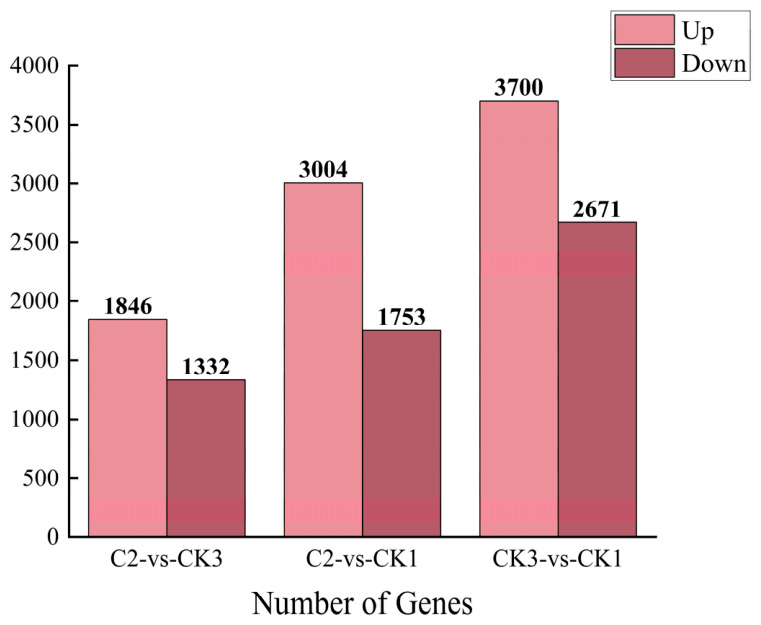
Quantitative statistics of differentially expressed genes among C2, CK3, and CK1 groups.

As shown in Figure 4, compared with CK3, C2 had 1846 up-regulated genes and 1332 down-regulated genes. Compared with the CK1 group, 3004 genes were up-regulated, and 1753 genes were down-regulated in the C2 group. Compared with the CK1 group, a total of 3700 genes were up-regulated, and 2671 genes were down-regulated in the CK3 group. In the pairwise comparison of the three groups, the number of up-regulated genes was significantly higher than that of down-regulated genes (*Q* < 0.05), indicating that the activation of gene expression was the main molecular regulation mode of alfalfa in response to cold plasma treatment and drought stress.

The sample differences in gene expression between the three groups of C2-vs.-CK3, C2-vs.-CK1, and CK3-vs.-CK1 are shown in Supplementary Figure S1. It was found that the differences between the multiples of the three gene groups were concentrated within ±5. The number of genes with up-regulated multiples greater than 5 in the three groups was significantly more than the number of genes with down-regulated multiples less than −5, which proved that compared with CK3 and CK1, the expression of C2 was significantly up-regulated. Compared with CK1, the expression of CK3 was significantly up-regulated, and the down-regulated genes were not significantly different among the three groups.

Among the differentially expressed genes, 20 genes with the largest up-regulated fold change in the treatment group were selected in each group, as shown in Table 2.

Gene expression across different treatment groups showed significant spatial and temporal specificity and functional differentiation. Among them, genes numbered 3, 6, 8, 9, 11, 12, 13, 15, 16 and 20 showed a very significant up-regulation trend in the C2 compared to CK3 (Qvalue < 0.05, log2FC > 0), while genes numbered 1, 2, 10, 14, 18 and 19 exhibited higher absolute expression levels in CK1 than in C2 and CK3 (based on FPKM values), indicating that the former was a stress-induced key gene and the latter was a basic growth maintenance gene. The two together constitute the balance of expression and regulation of cells between normal growth and stress adaptation; genes No. 3, 5, and 20 were not expressed in CK3. Genes No. 4, 13, 15, 16, and 20 were highly expressed in C2 and were almost silent in CK3. It was further confirmed that these genes were stress-specific response factors, which were activated and transcribed only in the presence of external stimuli, and were the core molecules that initiated the stress defense pathway. Based on Gene Ontology (GO) annotations and functional databases, genes 4, 13, and 20 are involved in extracellular signal transduction to the nucleus. They regulate a series of core life activities, such as cell division, differentiation, programmed cell death, and stress adaptation, by mediating signal perception, transduction, and amplification. As key components in maintaining genome stability, the No. 12 and 15 genes play an irreplaceable role in improving cell resistance to extreme stresses such as ionizing radiation and in participating in DNA damage repair and cell survival regulation. The above results are presented together, and the differentially expressed genes participate in the cell stress response through three mechanisms: signal transduction pathway activation, stress defense response initiation, and genome stability maintenance. They play a central role in the molecular network for cell perception of stress, signal transmission, initiation of protection procedures, and ultimately determination of cell fate. It provides an important candidate gene and theoretical basis for in-depth analysis of the regulation mechanism of alfalfa stress resistance.

Among the differentially expressed genes, 20 genes with the largest down-regulated fold change in the treatment group were identified in each group, as shown in Table 3.

The expression patterns of the C2 and CK3 groups were significantly different. Among them, genes No. 1, 3, 7, 13, and 14 were not detected in the C2 group, indicating that these genes may be completely silenced or specifically not expressed under this treatment condition. On the whole, all the genes numbered 1–20 showed a significant downward trend in the C2 group, indicating that their transcription levels were significantly inhibited. The function of No. 6, 12, and 13 genes is closely related to the regulation of endoplasmic reticulum homeostasis. Studies have shown that the abnormal accumulation of these gene-encoded products can induce endoplasmic reticulum stress and further initiate the unfolded protein response (UPR) signaling pathway to help cells restore protein folding and endoplasmic reticulum function homeostasis. However, when the stress intensity exceeds the cell’s own repair ability, the protective effect of UPR will not be sufficient to maintain normal physiological functions, which will eventually trigger the apoptosis process and lead to cell death. The above results suggest that these genes may play key biological functions in response to processing by regulating the endoplasmic reticulum stress-UPR pathway.

### 3.4. KEGG Pathway Enrichment Analysis

**Figure 5:**
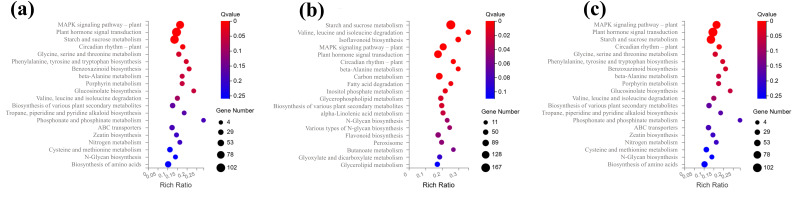
Gene expression KEGG enrichment bubble diagram of: (**a**) C2-vs.-CK3; (**b**) C2-vs.-CK1; (**c**) CK3-vs.-CK1.

KEGG Pathway enrichment analysis was performed on the differential genes in the C2 group and the CK3 group. The results are shown in Figure 5a. The up-regulated genes were significantly enriched in the plant MAPK signaling pathway, plant hormone signal transduction pathway, and plant circadian rhythm pathway (*Q* < 0.05). The numbers of enriched genes were 79, 102, and 90, and the enrichment factors were 0.21, 0.25, and 0.19, respectively. The synergistic activation of the above pathways can promote the translation of stress-related RNAs and the synthesis of functional proteins by regulating stress signal transduction, endogenous hormone synthesis and response, and rhythmic cellular metabolism. It is the core molecular pathway for cold plasma treatment to strengthen drought stress response. At the metabolic pathway level, starch and sucrose metabolism, porphyrin metabolism, and glycine-serine-threonine metabolism were significantly enriched up-regulated metabolic pathways (*Q* < 0.05). The enrichment factors were 0.14, 0.17, and 0.15, respectively. The numbers of enriched genes were 68, 41, and 37, respectively, indicating that cold plasma treatment can activate carbon-based cellular metabolism and nitrogen-containing substance metabolism, providing energy and material support for seed germination and seedling growth under drought stress.

The down-regulated genes were significantly enriched in the phosphate and hypophosphate metabolism pathway and N-glycosylation biosynthesis pathway (*Q* < 0.05). The number of enriched genes was 26 and 11, and the enrichment factors were 0.32 and 0.29, respectively. The above pathways are involved in tRNA processing and protein post-translational modification. Down-regulation of these genes can reduce the folding and synthesis consumption of non-essential proteins, redirect cellular metabolic resources to the synthesis of drought-related functional proteins, and improve the efficiency of resource allocation. Compared with the CK3-vs.-CK1 group, the number of up-regulated genes in the MAPK signaling pathway in the C2-vs.-CK3 group decreased by 61.4%, but the enrichment factor increased by 0.08, and the Q value decreased by 0.023, indicating that cold plasma treatment can specifically activate the core genes of the MAPK signaling pathway in alfalfa under drought stress, rather than broad-spectrum up-regulated pathway-related genes. This regulatory model can reduce energy consumption associated with invalid gene expression, which is a key molecular mechanism for improving alfalfa drought resistance.

The differential genes of C2 and CK1 were subjected to KEGG enrichment analysis. The results are shown in Figure 5b. The significant enrichment pathways of up-regulated genes were still dominated by stress resistance and signal transduction pathways. The number of enriched genes in the plant MAPK signaling pathway, plant hormone signal transduction pathway, and plant circadian rhythm pathway was 99, 122, and 30, respectively (*Q* < 0.05). The enrichment factors were 0.26, 0.29, and 0.11, respectively, which were higher than those in the C2-vs.-CK3 group, indicating that the synergistic effect of drought stress and cold plasma treatment could further strengthen the activation of the stress resistance signaling pathway. Enhance the cell’s ability to perceive and respond to adversity. In the metabolic pathway, starch and sucrose metabolism, β-alanine metabolism, carbon metabolism, and inositol phosphate metabolism were the core up-regulated pathways (*Q* < 0.05). The enrichment factors were 0.25, 0.29, 0.18, and 0.21, and the number of enriched genes was 89, 33, 156, and 29, respectively. The synergistic activation of the above pathways can accelerate the decomposition and transport of carbohydrates and promote the synthesis of osmoregulatory precursors, which not only provide energy for stress response but also lay a material foundation for cell osmoregulation. The down-regulated genes were significantly enriched in glyoxylic acid and dicarboxylic acid metabolic pathways and glycerolipid metabolic pathways (*Q* < 0.05). The number of enriched genes was 28 and 36, and the enrichment factors were 0.35 and 0.27, respectively. The glyoxylic acid cycle is the key pathway of photorespiration and lipid decomposition. Its gene down-regulation can reduce energy loss and oxidative damage caused by photorespiration, inhibit basal lipid decomposition, and maintain the stability of cell membrane structure. The down-regulation of the glycerolipid metabolic pathway can reduce the energy input of membrane lipid synthesis, improve the dehydration tolerance of the cell membrane by adjusting the composition of membrane lipid, induce the synthesis of lipid signaling molecules, activate ABA-mediated stomatal closure and other drought resistance responses, and ultimately improve the drought adaptability of alfalfa.

The differential genes of the CK3 group and CK1 group were analyzed by KEGG enrichment analysis. The results are shown in Figure 5c. Drought stress can independently activate the stress-related pathways of alfalfa. Plant MAPK signaling pathway, plant hormone signal transduction pathway, and plant circadian rhythm pathway are the core enrichment pathways of up-regulated genes (*Q* < 0.05). The number of enriched genes was 153, 179, and 90, respectively, and the enrichment factors were 0.13, 0.17, and 0.19, respectively, which were the basic molecular responses of alfalfa to drought stress. At the metabolic pathway level, starch and sucrose metabolism, β-alanine metabolism, inositol phosphate metabolism, and sphingolipid metabolism were significantly up-regulated (*Q* < 0.05). The enrichment factors were 0.33, 0.35, 0.28, and 0.33, respectively. The number of enriched genes was 118, 36, 45, and 52, respectively, indicating that alfalfa under drought stress can activate carbon metabolism and osmotic regulation-related metabolism to initiate stress response independently, but the number of enriched genes is large, and some pathway enrichment factors are high, reflecting its basic stress resistance regulation model.

There is also a problem with broad-spectrum activation and large resource consumption. The down-regulated genes were significantly enriched in the pyruvate, sulfur, and carotenoid biosynthesis pathways (*Q* < 0.05). The number of enriched genes was 42, 15, and 19, respectively, and the enrichment factors were 0.28, 0.31, and 0.26. Downregulation of pyruvate metabolism can reduce energy consumption during basal respiratory metabolism and redirect carbon flow toward the synthesis of osmoregulatory substances such as proline and soluble sugars. The down-regulation of sulfur metabolism can give priority to the synthesis of antioxidant sulfur-containing substances, such as glutathione, and reduce the consumption of non-essential sulfur-containing amino acids. The down-regulation of the carotenoid biosynthesis pathway can avoid the energy waste of photosynthetic pigment synthesis and reduce photooxidation damage. The three synergistically optimize the metabolic resources under drought stress, but the regulation efficiency is much lower than the specific regulation of the cold plasma treatment group.

### 3.5. Go Enrichment Analysis

**Figure 6:**
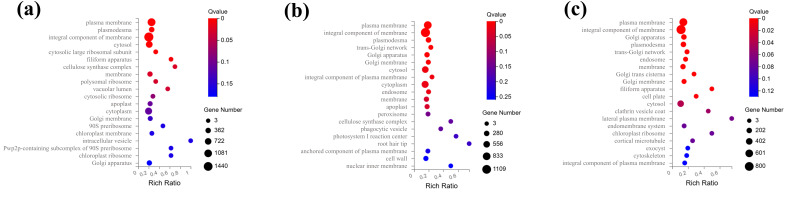
Gene expression GO enrichment bubble diagram of: (**a**) C2-vs.-CK3; (**b**) C2-vs.-CK1; (**c**) CK3-vs.-CK1.

As shown in Figure 6a, in the C2-vs.-CK3 comparison group, the up-regulated genes were significantly enriched in multiple biological process items related to drought stress response. The items of response to ‘water shortage’ were significantly enriched, including 52 up-regulated genes, active oxygen metabolism process’ contained 41 up-regulated genes, ‘abscisic acid activation signaling pathway’ contained 29 up-regulated genes, and ‘osmotic regulation’ contained 33 up-regulated genes (*Q* < 0.05). The enrichment results at the BP level above showed that cold plasma treatment enhanced the perception, signal transduction, and defense response of alfalfa seedlings to drought stress, which was consistent with the observation results of increased antioxidant enzyme activity and decreased malondialdehyde content in physiological indicators. At the molecular function level, the up-regulated genes were significantly enriched in ‘antioxidant activity’, including 58 up-regulated genes, ‘peroxidase activity’, including 27 up-regulated genes, ‘superoxide dismutase activity’, including 19 up-regulated genes, and ‘aquaporin activity’, including 12 up-regulated genes (*Q* < 0.05).

The enrichment results at the Molecular Function (MF) level provided molecular evidence for the up-regulation of antioxidant enzyme coding genes and water transport-related gene expression by cold plasma treatment, which was consistent with the physiological observation results of SOD, POD, and CAT activity increase and cell water balance maintenance. At the cellular component level, the up-regulated genes were mainly located in the ‘plasma membrane’, including 582 up-regulated genes, the ‘integral membrane’ contained 800 up-regulated genes, the ‘Golgi apparatus’ contained 143 up-regulated genes, and the ‘plasmodesmata’ contained 155 up-regulated genes (*Q* < 0.05). The above results indicate that cold plasma treatment enhances the structural integrity and intercellular communication efficiency of the membrane system, providing a structural basis for material transport and signal transduction.

The down-regulated genes were less enriched in the C2-vs.-CK3 comparison group, and only 22 down-regulated genes were included in the ‘integral component of membrane’ (*Q* < 0.05). The down-regulated gene set contained multiple aquaporin-coding genes, and the down-regulated expression indicated that reducing the water permeability of the cell membrane slowed down the water absorption in the early stage of germination, which was helpful for seedlings to establish a more robust defense system under drought stress.

As shown in Figure 6b, in the C2-vs.-CK1 comparison group, the GO enrichment pattern was similar to that of C2-vs.-CK3 but with a higher degree of enrichment. In the biological process, ‘response to abscisic acid’ was significantly enriched, including 67 up-regulated genes, ‘active oxygen scavenging’ contained 52 up-regulated genes, and ‘osmotic pressure regulation’ contained 48 up-regulated genes (*Q* < 0.05). At the molecular functional level, ‘oxidoreductase activity’ was significantly enriched, including 132 up-regulated genes, and ‘glutathione transferase activity ‘contained 23 up-regulated genes (*Q* < 0.05), indicating that cold plasma treatment activated multiple antioxidant defense mechanisms. In the cell component, except for the membrane system-related items, the ‘cell wall’ related genes were significantly down-regulated, including 126 down-regulated genes (*Q* < 0.05). These cell wall-related genes mainly include expansin and cellulose synthase family members. The down-regulation of its expression indicated that cold plasma treatment combined with drought stress induced a growth inhibition response, and preferentially allocated limited resources to osmotic regulation and antioxidant defense by reducing the energy consumption of cell wall synthesis and remodeling.

As shown in Figure 6c, the GO enrichment results of the CK3-vs.-CK1 comparison group reflected the basic molecular response of alfalfa to drought stress. In the biological process, ‘translation’ was significantly enriched, including 104 up-regulated genes, and ‘peptide biosynthesis’ contained 89 up-regulated genes (*Q* < 0.05), indicating that cells under drought stress initiate stress protein synthesis by enhancing translation activity. In the molecular function, ‘structural molecular activity’ was significantly enriched, including 76 up-regulated genes, and ‘ribosomal structural component’ contained 40 up-regulated genes (*Q* < 0.05), which was consistent with Biological Process (BP) results.

Among the cellular components, ‘cytoplasmic ribosomal large subunit’ was significantly enriched, containing 40 up-regulated genes, and ‘integral membrane component’ contained 1440 up-regulated genes (*Q* < 0.05). At the same time, the Golgi membrane was down-regulated, including 46 down-regulated genes (*Q* < 0.05). The enrichment pattern showed that when drought stress acted alone, plants mainly responded to stress by enhancing the protein synthesis machine, but the inhibition of the secretory pathway (Golgi apparatus) was weak, which was significantly different from the ‘precise activation and resource redirection’ pattern presented in the cold plasma treatment group.

### 3.6. qRT-PCR Verification

**Figure 7:**
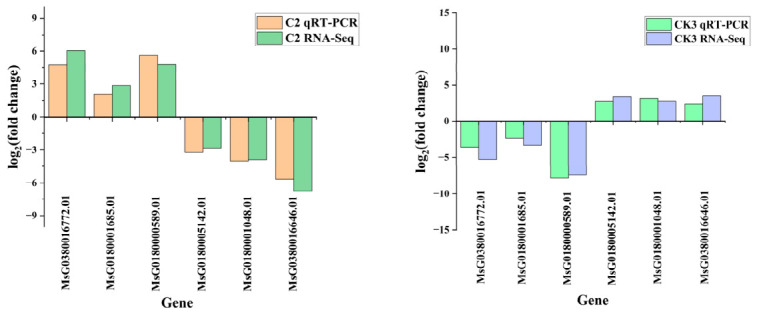
qRT-PCR verification of differentially expressed genes.

The results of qRT-PCR verification are shown in Figure 7. Three up-regulated genes and three down-regulated genes were randomly selected. Compared with the RNA-Seq results, these genes were differentially expressed, and the trend of differential expression was consistent, indicating that the analysis results of differentially expressed genes by the RNA-Seq method were reliable.

## 4. Discussion

### 4.1. Effects of Cold Plasma Treatment on Germination Index of Alfalfa Seeds Under Drought Stress

This study found that under different concentrations of PEG-6000 simulated drought stress, cold plasma treatment could significantly improve the germination potential, germination rate, germination index, and vitality index of alfalfa seeds, which was consistent with the research conclusions of Feng, J.K et al. [52], and each index showed an overall upward trend with the increase of treatment voltage. Among them, the 10% PEG-6000, 15 kV treatment group had the best effect, which was closely related to the fact that the seeds themselves did not reach the irreversible damage threshold under moderate drought stress, and the resistance potential was easily induced and activated by external mild stimulation. The effect of low voltage (5 kV, 10 kV) treatment was weaker than that of 15 kV. It was speculated that the lack of active signal intensity caused by discharge did not fully break seed dormancy and activate germination-related metabolism, which provided a theoretical basis and technical reference for improving its drought resistance and germination ability.

### 4.2. Effects of Cold Plasma Treatment on Physiological and Biochemical Indexes of Alfalfa Seeds Under Drought Stress

In this study, it was found that the activities of SOD, POD, and CAT in the 15 kV cold plasma treatment group were significantly higher than those in the control group (*p* < 0.05), and the content of MDA was significantly lower (*p* < 0.05), which was consistent with the research results of Li, L. et al. [58] and Li, L. et al. [48] also reported that cold plasma treatment enhances antioxidant enzyme activities and alleviates oxidative stress. This is because the Reactive Oxygen and Nitrogen Species (RONS) produced by cold plasma discharge, as a mild stress signal, can induce the early activation of the antioxidant system in plants. As the first line of defense in the antioxidant system, SOD can rapidly remove superoxide anions, while POD and CAT further decompose hydrogen peroxide, thereby minimizing membrane lipid peroxidation and reducing MDA accumulation. The three formed an antioxidant defense network with significant positive intercorrelations among them (*p* < 0.05) and negative correlations with MDA (*p* < 0.05), which effectively alleviated the oxidative damage under drought stress, and the decrease of MDA content directly proved that cold plasma treatment could significantly inhibit the membrane lipid peroxidation caused by drought [59], maintain the integrity of seedling cells, provide structural guarantee for the transportation of water and nutrients, and enhance the drought resistance of alfalfa in arid and semi-arid areas [48,58].

### 4.3. Effects of Cold Plasma Treatment on Drought Resistance Gene Expression of Alfalfa Seeds Under Drought Stress

KEGG pathway enrichment showed that after cold plasma treatment, the up-regulated genes of the MAPK signaling pathway, plant hormone signal transduction, starch and sucrose metabolism, and other stress resistance pathways in alfalfa seedlings were dominant. Cold plasma treatment promoted the up-regulation of MAPK pathway genes, so that they could quickly transmit drought signals and activate stress-resistant genes. At the same time, it accelerated the plant hormone signaling pathway to regulate hormone synthesis and conduction, and optimized stomatal movement and root growth. Enrichment of starch and sucrose metabolism can quickly supply energy and alleviate energy deficit, which together constitute the drought molecular adaptation mechanism of alfalfa [60].

GO functional enrichment showed that cold plasma could regulate differential gene expression in alfalfa seeds and seedlings, consistent with the research results of Xu, F. et al. [25] The differential genes were enriched in stress-related pathways and functional elements, and coordinated with the changes of physiological and biochemical indexes in the early stage, which verified the consistency of the molecular mechanism and physiological response. In addition, the core drought-resistant genes identified among differentially expressed genes with high specificity in the 15 kV treatment group can enhance the drought adaptability of plants and provide the molecular basis and a reference for drought-resistant breeding and the application of cold plasma in alfalfa.

### 4.4. Limitations and Future Prospects

This study only performed single-factor optimization of cold plasma voltage and did not involve orthogonal synergistic regulation of treatment time, discharge power, and other parameters.

In the future, the multi-factor orthogonal test of cold plasma treatment voltage, time, and discharge power will be carried out, and the optimal treatment system will be constructed by combining the response surface method, which will lay a theoretical and practical foundation for the wide application of plasma agriculture in the field of agricultural stress resistance.

## 5. Conclusions

In this study, alfalfa was used as the material, and 0, 5, 10, 15 kV discharge plasma treatment for 5 min, combined with 0–15% PEG-6000 simulated gradient drought stress, was used to systematically explore the regulation effect and mechanism of cold plasma on drought resistance of alfalfa from seed germination, seedling physiology and biochemistry, and gene transcription level. It was clear that cold plasma of 15 kV and 5 min was the optimal treatment parameter to improve its drought resistance, which could significantly improve the germination indexes, such as seed germination potential and germination rate (*p* < 0.05). The activities of antioxidant enzymes such as SOD, POD, and CAT in seedlings were up-regulated, and the content of MDA was decreased (*p* < 0.05), which effectively inhibited the damage of membrane lipid peroxidation. At the same time, the expression of stress-related pathway genes, such as the MAPK signaling pathway and plant hormone signal transduction, was activated, and the metabolic pathway was regulated to achieve energy and carbon source redirection, forming a drought-resistant response mode to strengthen stress defense and optimize resource allocation, so as to improve the drought adaptability of alfalfa seeds and seedlings from multiple dimensions.

## Figures and Tables

**Table 1:** Stress concentration and treatment intensity parameters.

Group	Concentration of PEG-6000 Solution/%	Processing Voltage/kV	Treatment Time/ Minute
CK1 ^1^	0	0	5
CK2	5	0	5
CK3	10	0	5
CK4	15	0	5
A1	5	5	5
A2	10	5	5
A3	15	5	5
B1	5	10	5
B2	10	10	5
B3	15	10	5
C1	5	15	5
C2	10	15	5
C3	15	15	5

Note: Subscript 1 refers to no plasma treatment, no drought stress.

**Table 2:** Significantly up-regulated genes after treatment.

Serial Number	Gene ID	C2 Average FPKM	CK3 Average FPKM	CK1 Average FPKM	log2 (C2/CK3)	Qvalue (C2/CK3)
1	MsG0180000589.01	403.92	13.91	1694.19	5.06	0.0000
2	MsG0280006340.01	18.48	0.58	73.33	5.22	0.0361
3	MsG0280006916.01	8.95	0.00	5.31	5.37	0.0215
4	MsG0280007716.01	2.20	0.04	0.05	5.62	0.0086
5	MsG0380015969.01	2.31	0.00	4.77	6.36	0.0006
6	MsG0380016772.01	150.31	2.60	59.78	6.04	0.0000
7	MsG0380017548.01	216.39	6.43	253.70	5.26	0.0000
8	MsG0480019805.01	1.36	0.07	0.01	4.50	0.0114
9	MsG0480020361.01	3.34	0.12	0.19	5.10	0.0300
10	MsG0480020754.01	5.63	0.29	13.71	4.56	0.0000
11	MsG0480021712.01	3.81	0.11	1.81	5.92	0.0095
12	MsG0480023413.01	1377.04	51.62	498.51	4.92	0.0000
13	MsG0480023440.01	5.49	0.20	0.81	5.74	0.0056
14	MsG0580025429.01	2.04	0.10	7.38	4.53	0.0109
15	MsG0580027087.01	3.86	0.02	0.02	7.98	0.0134
16	MsG0580027126.01	3.01	0.09	0.43	5.30	0.0000
17	MsG0580027554.01	0.72	0.02	0.05	6.19	0.0150
18	MsG0580027607.01	36.17	1.58	87.24	4.77	0.0006
19	MsG0580027714.01	6.50	0.13	13.12	6.25	0.0003
20	MsG0780037353.01	4.24	0.00	0.93	5.53	0.0253

**Table 3:** Significantly down-regulated genes after treatment.

Serial Number	Gene ID	C2 Average FPKM	CK3 Average FPKM	CK1 Average FPKM	log2 (C2/CK3)	Qvalue (C2/CK3)
1	MsG0080047990.01	0.00	1.44	0.27	−5.39	0.0198
2	MsG0180000226.01	0.32	10.68	5.16	−4.91	0.0004
3	MsG0180000903.01	0.00	13.81	0.00	−23.67	0.0000
4	MsG0180002953.01	0.03	0.93	0.13	−4.83	0.0299
5	MsG0280010209.01	0.98	22.78	0.33	−4.35	0.0003
6	MsG0480019308.01	2.93	79.11	5.71	−4.62	0.0421
7	MsG0480019653.01	0.00	0.70	0.04	−4.90	0.0461
8	MsG0480022820.01	0.40	9.85	0.00	−4.45	0.0089
9	MsG0480022904.01	0.33	7.28	9.59	−4.75	0.0079
10	MsG0480023344.01	0.97	33.43	0.34	−4.98	0.0092
11	MsG0680032476.01	1.85	76.80	0.00	−5.23	0.0042
12	MsG0680032479.01	3.33	97.74	0.00	−4.64	0.0036
13	MsG0680032603.01	0.00	28.35	0.00	−6.10	0.0019
14	MsG0680035667.01	0.00	1.45	2.21	−5.33	0.0188
15	MsG0780037521.01	0.61	13.26	0.15	−4.31	0.0043
16	MsG0780038694.01	0.08	1.67	0.05	−4.43	0.0054
17	MsG0880041928.01	0.77	20.07	0.19	−4.58	0.0295
18	MsG0880044731.01	0.31	7.74	7.67	−4.50	0.0072
19	MsG0880046039.01	0.38	10.61	0.19	−4.70	0.0098
20	MsG0880047320.01	0.41	11.72	0.07	−4.68	0.0000

## Data Availability

The raw sequence data reported in this paper have been deposited in the Genome Sequence Archive (Genomics, Proteomics & Bioinformatics 2025) in the National Genomics Data Center (Nucleic Acids Res 2026), China National Center for Bioinformation/Beijing Institute of Genomics, Chinese Academy of Sciences [61,62] (GSA: CRA043195) that are publicly accessible at https://ngdc.cncb.ac.cn/gsa/browse/CRA043195 (accessed on 13 April 2026).

## Supplementary Materials

The following supporting information can be downloaded at: https://www.mdpi.com/article/10.3390/antiox15060681/s1. Figure S1: Sample difference volcano map: (a) C2-vs.-CK3; (b) C2-vs.-CK1; (c) CK3-vs.-CK1.

## Abbreviations

The following abbreviations are used in this manuscript:

MDA	Malondialdehyde
POD	Directory of open access journals
SOD	Superoxide Dismutase
CAT	Catalase
NBT	Nitroblue tetrazolium chloride
TBA	Thiobarbituric acid
*p* value	Probability Value
Q value	Corrected Probability Value
FPKM	Fragments Per Kilobase of transcript per Million mapped reads
MAPK	Mitogen-Activated Protein Kinase
PEG-6000	Polyethylene Glycol 6000
RONS	Reactive Oxygen and Nitrogen Species
BP	Biological Process
MF	Molecular Function
UPR	Unfolded Protein Response
RNA-Seq	RNA Sequencing
qRT-PCR	Quantitative Real-Time Polymerase Chain Reaction
PBS	Phosphate Buffer Solution

## References

[1] Eziz A.W., Yan Z.B., Tian D., Han W.X., Tang Z.Y., Fang J.Y. (2017). Drought effect on plant biomass allocation: A meta-analysis. Ecol. Evol..

[2] Yu J., Jiang M.Y., Guo C.K. (2019). Crop pollen development under drought: From the phenotype to the mechanism. Int. J. Mol. Sci..

[3] Samarah N.H. (2005). Effects of drought stress on growth and yield of barley. Agron. Sustain. Dev..

[4] Zipper S.C., Qiu J.X., Kucharik C.J. (2016). Drought effects on US maize and soybean production: Spatiotemporal patterns and historical changes. Environ. Res. Lett..

[5] Sabagh A.E., Hossain A., Barutular C., Gormus O., Ahmad Z. (2019). Effects of drought stress on the quality of major oilseed crops: Implications and possible mitigation strategies—A review. Appl. Ecol. Environ. Res..

[6] Cohen I., Zandalinas S.I., Huck C., Fritschi F.B., Mittler R. (2020). Meta-analysis of drought and heat stress combination impact on crop yield and yield components. Physiol. Plant..

[7] Ullah A., Farooq M. (2022). The challenge of drought stress for grain legumes and options for improvement. Arch. Agron. Soil. Sci..

[8] Yin X.W., Feng Q., Li Y., Liu W., Zhu M., Zhang J.T., Yang L.S., Zhang C.Q., Wu X., Zheng X.J. (2022). Exacerbated drought accelerates catastrophic transitions of groundwater-dependent ecosystems in arid endorheic basins. J. Hydrol..

[9] Zhu X.Y., Huang S.Z., Singh V.P., Huang Q., Zhang H.B., Leng G.Y., Gao L., Li P., Guo W.W., Peng J. (2025). Terrestrial ecosystem resilience to drought stress and driving mechanisms thereof in the yellow river basin, China. J. Hydrol..

[10] He M.Z., Dijkstra F.A. (2014). Drought effect on plant nitrogen and phosphorus: A meta-analysis. New Phytol..

[11] Todaka D., Shinozaki K., Yamaguchi-Shinozaki K. (2015). Recent advances in the dissection of drought-stress regulatory networks and strategies for development of drought-tolerant transgenic rice plants. Front. Plant Sci..

[12] Dormatey R., Sun C., Ali K., Coulter J.A., Bi Z.Z., Bai J.P. (2020). Gene pyramiding for sustainable crop improvement against biotic and abiotic stresses. Agron. J..

[13] Li J.Y., Liu H.W., Wang J.T., Macdonald C.A., Singh P., Cong V.T., Klein M., Delgado-Baquerizo M., Singh B.K. (2025). Drought-induced plant microbiome and metabolic enrichments improve drought resistance. Cell Host Microbe.

[14] Zhu X.Y., Chen W.X., Li M.Y., Liu B.S., Zhao S.M., Hu M.L., Li J.J., Li D.F., Shi Y.H., Sun H. (2025). Comprehensive evaluation of the nutritional value and contaminants of alfalfa (*Medicago sativa* L.) in China. Front. Nutr..

[15] Zhang Y.Y., Wang L. (2025). Advances in basic biology of alfalfa (*Medicago sativa* L.): A comprehensive overview. Hortic. Res..

[16] Wang Y., Long S.S., Zhang J.Y., Wang P.C., Zhao L.L. (2025). Evaluation of growth, physiological, and biochemical responses of different *Medicago sativa* L. Varieties under drought stress. Plants.

[17] Wang W.J., Kang W.J., Shi S.L., Liu L.B. (2025). Widely targeted metabolic profiling reveals drought resistance mechanisms in alfalfa leaves. BMC Plant Biol..

[18] Peng W.X., Cai W.Q., Pan J.Y., Su X.R., Dou L.R. (2025). Molecular mechanisms of alfalfa response to abiotic stresses. Plants.

[19] Shi S.L., Nan L.L., Smith K.F. (2017). The Current Status, Problems, and Prospects of Alfalfa (*Medicago sativa* L.) Breeding in China. Agronomy.

[20] Sima N.A.K., Jabbari H., Ebadi A., Ghaffari M.R., Koobaz P. (2023). Comparative Analysis of Exogenous Hormone Application on Contrasting Canola (*Brassica napus* L.) Genotypes Under Drought Stress Conditions. J. Soil. Sci. Plant Nutr..

[21] Lv H.L., Jiang Y.B., Qi G.P., Yin M.H., Kang Y.X., Ma Y.L., Wang Y.Y., Xiao F., Peng J.Q., Li H.Y. (2025). Effects of Water-Nitrogen Management on the Growth and Nitrogen Uptake and Utilization of Intercropped Alfalfa. Plants.

[22] Li L., Chen J.B., Wang H.R., Guo H.L., Li D.D., Li J.J., Liu J.Q., Shao H.L., Zong J.Q. (2021). Cold plasma treatment improves seed germination and accelerates the establishment of centipedegrass. Crop Sci..

[23] Sirgedaite-Seziene V., Lucinskaite I., Mildaziene V., Ivankov A., Koga K., Shiratani M., Lauzike K., Baliuckas V. (2022). Changes in content of bioactive compounds and antioxidant activity induced in needles of different half-sib families of norway spruce ((l.) h. Karst) by seed treatment with cold plasma. Antioxidants.

[24] Karimi J., Bansal S.A., Kumar V., Pasalari H., Badr A.A., Nejad Z.J. (2024). Effect of cold plasma on plant physiological and biochemical processes: A review. Biologia.

[25] Xu F., Chen H., Chen C., Liu J.Q., Song Z.Q., Ding C.J. (2024). The mutagenic effect of cold plasma on *Medicago sativa* L.. Free Radic. Biol. Med..

[26] Shu J.W., Xu X.Q., Cheng H., Cui Y.H., Ni J.P., Ni C.S. (2024). Direct nitrogen fixation in air over soil using dielectric barrier discharge plasma for enhanced plant growth. Plasma Process. Polym..

[27] Li Y.B., Song Z.Q., Zhang T., Ding C.J., Chen H. (2022). Gene expression variation of astragalus adsurgens pall. Through discharge plasma and its activated water. Free Radic. Biol. Med..

[28] Ilyas M., Nisar M., Khan N., Hazrat A., Khan A.H., Hayat K., Fahad S., Khan A., Ullah A. (2020). Drought tolerance strategies in plants: A mechanistic approach. J. Plant Growth Regul..

[29] Yang Z.R., Qin F. (2023). The battle of crops against drought: Genetic dissection and improvement. J. Integr. Plant Biol..

[30] Cao Y.Y., Yang W.B., Ma J., Cheng Z.Q., Zhang X., Liu X.M., Wu X.L., Zhang J.H. (2024). An integrated framework for drought stress in plants. Int. J. Mol. Sci..

[31] Choe H.C., Ri S.H., Kim J.P., Ri I.S., Pak S.C., Kim Y.J. (2025). Gliding arc discharge plasma treatment for promoting germination of wheat seed at low ambient temperature. J. Vac. Sci. Technol. A.

[32] Shukurov O., Rasulov S., Kodirov A., Karimov H., Hamdamov J., Norbutayeva B., Azimova N., Attri P., Shiratani M., Guo L. (2025). Cold atmospheric plasma for eco-friendly wheat production: Regional case studies from uzbekistan. Plasma Process. Polym..

[33] Esmaeili F., Ramezani Kaporchali M., Razavi K., Ahmadi M., Hejri S., Begri M., Naeimabadi A., Lohrasebi T. (2026). Highlighting the cold plasma effect on wheat performance: Enhancing drought tolerance, and improving baking quality. Curr. Plant Biol..

[34] Zhang M.J., Song Z.Q., Li B.F., Qin C.X., Ding C.J., Liu L.Q. (2025). Enhancing drying efficiency and nutritional quality of oat grass using high-voltage discharge plasma drying. Chem. Biol. Technol. Agric..

[35] Zhang M.J., Song Z.Q., Li B.F., Qin C.X., Ding C.J., Liu L.Q. (2025). Study on the effects of high-voltage discharge plasma drying on the volatile organic compounds and texture characteristics of oat grass. Agriculture.

[36] Wang X.X., Liu Z.D., Zhao P.S., Song Z.Q. (2025). Effects of high-voltage discharge plasma on drying properties, microstructure, and nutrients of oat grass. Agron. J..

[37] Hashizume H., Kitano H., Mizuno H., Abe A., Yuasa G., Tohno S., Tanaka H., Ishikawa K., Matsumoto S., Sakakibara H. (2020). Improvement of yield and grain quality by periodic cold plasma treatment with rice plants in a paddy field. Plasma Process. Polym..

[38] Yang W.C., Ma H.H., Lan H.L., Liao W.X., Lin X.Y., Li C. (2025). Application of cold plasma for rice bran preservation: Effects on stability and quality. Innov. Food Sci. Emerg. Technol..

[39] Yang X.N., Ma L.X., Li X.N., Bai J.W., Zhou R.Y., Wang C., Cai J.R. (2026). Cold plasma enhances brown rice storage stability: Discharge modes, uniformity, and energy deposition in air and argon atmospheres. Food Chem..

[40] Jin Z.M., Ren Y.F., Wan Y., Ouyang D., Zhang H., Huang K., Sang S.Y., Liu Y.N., Xing J.L., Luo X.H. (2026). Effect of dielectric barrier discharge cold plasma treatment on high moisture brown rice and its starch characteristics. Food Bioprocess Technol..

[41] Chen G.Y., Dong S., Zhao S., Li S.H., Chen Y. (2019). Improving functional properties of zein film via compositing with chitosan and cold plasma treatment. Ind. Crops Prod..

[42] Grbic J.Z., Mladenovic D.D., Veljkovic M.B., Lazarevic S.S., Levic S.M., Lazovic S.S., Djukic-Vukovic A.P. (2024). Cold plasma/alkaline pretreatment facilitates corn stalk fractionation and valorization towards zero-waste approach. Ind. Crops Prod..

[43] Singh M., Vajpayee M., Ledwani L., Nema S.K. (2024). Synergistic surface treatment of corn fabric using dielectric barrier discharge plasma and plant extracts for enhancing antibacterial performance. Ind. Crops Prod..

[44] Adhikari B., Adhikari M., Ghimire B., Adhikari B.C., Park G., Choi E.H. (2020). Cold plasma seed priming modulates growth, redox homeostasis and stress response by inducing reactive species in tomato (*Solanum lycopersicum*). Free Radic. Biol. Med..

[45] Li K., Zhong C.S., Shi Q.H., Bi H.G., Gong B. (2021). Cold plasma seed treatment improves chilling resistance of tomato plants through hydrogen peroxide and abscisic acid signaling pathway. Free Radic. Biol. Med..

[46] Su Z.L., Dong Z.D., Zhou Z.K., Sun B.J., Yang K., Liang J.P., Chang D.L., Yang D.Z. (2025). A study on the effect of dielectric barrier discharge on the germination and surface characteristics of tomato seeds. Plasma Process. Polym..

[47] Salami S., Ghorbani A., Rostami M., Mazandarani A., Koolivand D. (2026). Sustainable cold plasma suppresses tomato brown rugose fruit virus in tomato seeds. Physiol. Mol. Plant Pathol..

[48] Li L., Zhang L., Dong Y.H. (2025). Seed priming with cold plasma mitigated the negative influence of drought stress on growth and yield of rapeseed (*Brassica napus* L.). Ind. Crops Prod..

[49] Wang Y.X., Zafar N., Ali Q., Manghwar H., Wang G.Y., Yu L., Ding X., Ding F., Hong N., Wang G.P. (2022). Crispr/cas genome editing technologies for plant improvement against biotic and abiotic stresses: Advances, limitations, and future perspectives. Cells.

[50] Chen L.F., Zhao Y., Zhu X.L., Wang Y.Z., Wang X., Wei X.H. (2026). Exogenous no enhances drought tolerance in alfalfa via the lignin synthesis pathway. BMC Genom..

[51] Wu B., Shi S.L., Kang W.J., Han Y.L. (2025). Genome-wide identification and expression pattern analysis under drought stress of phd family genes in alfalfa (*Medicago sativa*). BMC Genom..

[52] Meng X., Yi D.X., Ma L., Ma X.R., Xie K.Y., Wang X.M., Yang J.B., Tang J. (2025). Genome-wide identification of the m6a gene family and analysis of m6a methylome in alfalfa under drought stress. Plant Physiol. Biochem..

[53] Feng J.K., Wang D.C., Shao C.Y., Zhang L.L., Tang X. (2018). Effects of cold plasma treatment on alfalfa seed growth under simulated drought stress. Plasma Sci. Technol..

[54] Mir R.A., Khah M.A. (2024). Recent progress in enzymatic antioxidant defense system in plants against different environmental stresses. Improving Stress Resilience in Plants.

[55] Zhang C.M., Shi S.L., Liu Z., Yang F., Yin G.L. (2019). Drought tolerance in alfalfa (*Medicago sativa* L.) varieties is associated with enhanced antioxidative protection and declined lipid peroxidation. J. Plant Physiol..

[56] Chaachouay N., Ansari M.K.A., Houssni M., Zidane L., Husen A. (2025). Enzymatic Antioxidant Defense Systems in Plants. Role of Antioxidants in Mitigating Plant Stress.

[57] Huang W.G., Shi D.M., Cheng A.H., Chen G.F., Liu F., Zhang X.B., Dong J.N., Lan J., Ren H.B., Guo W. (2026). The responses of rice plant to tricyclazole at the transcriptome and metabolome levels. Front. Plant Sci..

[58] Li L., Li J.G., Shen M.C., Zhang C.L., Dong Y.H. (2015). Cold plasma treatment enhances oilseed rape seed germination under drought stress. Sci. Rep..

[59] Shahabi Z.M., Nasibi F., Noori H. (2025). Cold plasma technology as a pre-treatment for seed priming enhances germination and reduces salinity stress in Prosopis Koelziana. Sci. Rep..

[60] Chen F.Q., Ha X., Ma T., Ma H.L. (2024). Comparative analysis of the physiological and transcriptomic profiles reveals alfalfa drought resistance mechanisms. BMC Plant Biol..

[61] CNCB–NGDC Members and Partners (2025). Database resources of the National Genomics Data Center, China National Center for Bioinformation in 2026. Nucleic Acids Res..

[62] Zhang S.S., Chen X., Jin E.H., Wang A.K., Chen T.T., Zhang X.L., Zhu J.W., Dong L.L., Sun Y.L., Yu C.X. (2025). The GSA Family in 2025: A Broadened Sharing Platform for Multi-Omics and Multimodal Data. Genom. Proteom. Bioinform..

