# Supplementary material for: Cold Plasma Treatment Enhances Drought Tolerance of Alfalfa (Medicago sativa L.) Seeds by Modulating Physiological Responses and Transcriptomic Profiles
Source: Antioxidants (Basel). 2026 May 28;15(6):681. doi: 10.3390/antiox15060681 (PMC13295987; doi:10.3390/antiox15060681)
Supplement: Supplementary file 1 [file antioxidants-15-00681-s001.zip › antioxidants-4288829-supplementary.pdf]

Cold plasma treatment enhances drought tolerance of alfalfa (*Medicago sativa* L.) seeds by modulating physiological responses and transcriptomic profiles

Weicheng Gong [1], Chunxu Qin [1,2,3],Zhiqing Song [1,2,3]*, Xiliang Hao[1] , Aozhe Li[1], Yaxin Liu[1], Chengzhi Ma[1]

College of Electric Power, Inner Mongolia University of Technology, Hohhot 010080, China; 202311211352@imut.edu.cn(W.G.); qincx@imut.edu.cn(C.Q.); zqsong@imut.edu.cn(Z.S.); 202311211019@imut.edu.cn(X.H.); 202311211392@imut.edu.cn(A.L.); 202311211332@imut.edu.cn(Y.L.); 202311211372@imut.edu.cn(C.M.)

2 Inner Mongolia Key Laboratory of Intelligent Control for New Energy Power Systems, Hohhot 010080, China;

3 Engineering Research Center of the Ministry of Education for Large Scale Energy Storage Technology, Hohhot 010080, China;

* Correspondence: zqsong@imut.edu.cn; Tel.: +86-471-3602300

1. Volcanic plot

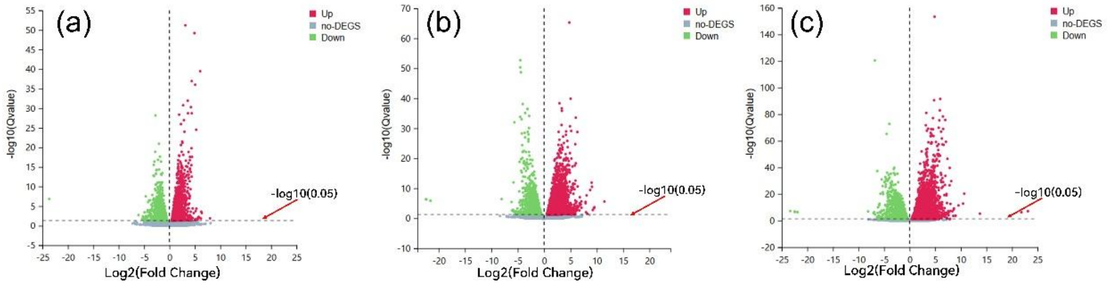

Supplementary figure S1. Sample difference volcano map:(a) C2-vs-CK3; (b) C2-vs-CK1; (c) CK3-vs-CK1.

2. Transcriptome sequencing raw data

The raw sequence data reported in this paper have been deposited in the Genome Sequence Archive (Genomics, Proteomics & Bioinformatics 2025) in the National Genomics Data Center (Nucleic Acids Res 2026), China National Center for Bioinformation/Beijing Institute of Genomics, Chinese Academy of Sciences[1,2] (GSA: CRA043195) that are publicly accessible at https://ngdc.cncb.ac.cn/gsa/browse/CRA043195.

References

1. CNCB–NGDC Members and Partners. Database resources of the National Genomics Data Center, China National Center for Bioinformation in 2026. Nucleic Acids Res. 2025, 54, D28–D47.
2. Zhang, S.S.; Chen, X.; Jin, E.H.; Wang, A.K.; Chen, T.T.; Zhang, X.L.; Zhu, J.W.; Dong, L.L.; Sun, Y.L.; Yu, C.X.; et al. The GSA Family in 2025: A Broadened Sharing Platform for Multi-Omics and Multimodal Data. Genom. Proteom. Bioinform. 2025, 23, qzaf072.
